# Advanced magnetic resonance imaging and neuropsychological assessment for detecting brain injury in a prospective cohort of university amateur boxers

**DOI:** 10.1016/j.nicl.2017.04.026

**Published:** 2017-04-26

**Authors:** M.G. Hart, C.R. Housden, J. Suckling, R. Tait, A. Young, U. Müller, V.F.J. Newcombe, I. Jalloh, B. Pearson, J. Cross, R.A. Trivedi, J.D. Pickard, B.J. Sahakian, P.J. Hutchinson

**Affiliations:** a Academic Division of Neurosurgery, Department of Clinical Neurosciences, Department of Neurosurgery, Box 167, Addenbrooke's Hospital, Cambridge CB2 0QQ, United Kingdom; b Department of Psychiatry, University of Cambridge and the MRC/Wellcome Trust Behavioural and Clinical Neuroscience Institute, Cambridge CB2 2QQ, United Kingdom; c Adult ADHD Service, Cambridgeshire and Peterborough NHS Foundation Trust, Cambridge Road, Fulbourn, Cambridge CB21 5HH, United Kingdom; d Wolfson Brain Imaging Centre, Addenbrooke's Hospital, Cambridge CB2 0QQ, United Kingdom; e University Division of Anaesthesia, University of Cambridge, Cambridge CB2 0QQ, United Kingdom; f Department of Radiology, Addenbrooke's Hospital, Cambridge CB2 0QQ, United Kingdom

**Keywords:** Boxing, Neuroimaging, Brain structure, CANTAB

## Abstract

**Background/aim:**

The safety of amateur and professional boxing is a contentious issue. We hypothesised that advanced magnetic resonance imaging and neuropsychological testing could provide evidence of acute and early brain injury in amateur boxers.

**Methods:**

We recruited 30 participants from a university amateur boxing club in a prospective cohort study. Magnetic resonance imaging (MRI) and neuropsychological testing was performed at three time points: prior to starting training; within 48 h following a first major competition to detect acute brain injury; and one year follow-up. A single MRI acquisition was made from control participants. Imaging analysis included cortical thickness measurements with Advanced Normalization Tools (ANTS) and FreeSurfer, voxel based morphometry (VBM), and Tract Based Spatial Statistics (TBSS). A computerized battery of neuropsychological tests was performed assessing attention, learning, memory and impulsivity.

**Results:**

During the study period, one boxer developed seizures controlled with medication while another developed a chronic subdural hematoma requiring neurosurgical drainage. A total of 10 boxers contributed data at to the longitudinal assessment protocol. Reasons for withdrawal were: logistics (10), stopping boxing (7), withdrawal of consent (2), and development of a chronic subdural hematoma (1). No significant changes were detected using VBM, TBSS, cortical thickness measured with FreeSurfer or ANTS, either cross-sectionally at baseline, or longitudinally. Neuropsychological assessment of boxers found attention/concentration improved over time while planning and problem solving ability latency decreased after a bout but recovered after one year.

**Conclusion:**

While this neuroimaging and neuropsychological assessment protocol could not detect any evidence of brain injury, one boxer developed seizures and another developed a chronic sub-dural haematoma.

## 1 Introduction

The sport of boxing is an emotive and contentious subject. Many medical and charitable organisations have called for an outright ban on both amateur and professional boxing. Campaigners against boxing have attracted widespread media coverage with articles in both the written press and broadcasting arena (McCabe, 2009; British Broadcasting Corporation (BBC), 1998). Counter-arguments from pro-boxing organisations emphasise the intensive medical monitoring of participants (ABAE, 2006) and a reduction in exposure to cumulative head injury with modern regulations (Clausen et al., 2005). There are also potential benefits of boxing on physical fitness and in providing a positive, disciplined training environment, which can be particularly valuable in socially deprived communities.

Despite the high level of scrutiny that boxing attracts, the scientific evidence that underpins regulation is far from comprehensive (Loosemore et al., 2007). A spectrum of brain injury has been described in boxers, ranging from post-concussive syndrome to chronic traumatic encephalopathy (CTE) and rare instances of life-threatening haemorrhage (Hart et al., 2012). Despite these methodological shortcomings, evidence suggests the development of CTE (or dementia pugilistica) in retired ex-professional fighters from the historical era of regulations (Roberts, 1969). Questions remain regarding why some participants develop CTE while others do not, whether some participants are able to tolerate repeated head injury, and if ultimately boxing has been made safe enough through modern regulations to prevent brain injury.

Modern neuroimaging techniques are an attractive tool to help improve our understanding of the pathophysiology involved in boxing related brain injury. Initially MRI was only used in boxing with small case series and limited analysis methods, which led to rather non-specific findings (Levin et al., 1987, Jordan and Zimmerman, 1988, Jordan and Zimmerman, 1990a, Haglund and Bergstrand, 1990, Holzgraefe et al., 1992, Hähnel et al., 2008, Hasiloglu et al., 2011). Increasingly sophisticated analysis methods of structural imaging data have been developed enabling measurement of grey matter thickness and density, subcortical volume changes, and white matter microstructure at the voxel level for the whole brain. Recent studies that have applied these methods to participants with concussion from a variety of different sports (predominantly American Football, Football, Ice Hockey, and Mixed Martial Arts) have revealed convergent trends for concussion exposure to be related to reduced cortical thickness (Koerte et al., 2015, Albaugh et al., 2015, Tremblay et al., 2013), smaller subcortical structure volumes (Bernick et al., 2015a, Singh et al., 2014), and altered white matter diffusion metrics (Lancaster et al., 2016, Meier et al., 2015, Stamm et al., 2015, Wilde et al., 2015, Tremblay et al., 2014, Shin et al., 2014, Bazarian et al., 2014, Hart et al., 2013, Virji-Babul et al., 2013). Additionally, these findings have often corresponded to impaired neuropsychological function but appear to be more persistent, suggesting a biomarker of longer-term structural alteration. Limitations of these studies often include unmatched control groups, cross-sectional or retrospective designs, and a lack of statistical power. Nevertheless, modern neuroimaging techniques now have an established sensitivity to detecting structural morphology alterations related to sports concussion.

We set out to test whether signs of brain injury could be found to develop over the short term in otherwise young healthy individuals taking up amateur boxing for the first time. In order to test for brain injury, we used an advanced magnetic resonance imaging (MRI) protocol and extensive battery of computerized neuropsychological tests. We planned to recruit participants from a local university amateur boxing club and follow them with three assessments (baseline, post-bout and one year follow-up) over approximately 18 months.

## 2 Materials & methods

### 2.1 Participants

Boxers were recruited from Cambridge University Amateur Boxing Club (CUABC). Recruitment began at the start of the academic year prior to commencing boxing training. All participants were members of the Amateur Boxing association of England (ABAE) and had undergone a satisfactory medical examination. Exclusion criteria included: any prior participation in boxing; any participation in contact sports (particularly martial arts or rugby) since age 18; any previous history of neurological disease, neurosurgery or psychiatric disorder; any history of claustrophobia; and any metal implants within the head or neck. Demographic information collected included age, gender and intelligence quotient (IQ) as measured by the National Adult Reading Test (NART).

Contemporaneous MRI from control participants, who were not participating in boxing, were included in a cross-sectional analysis at baseline.

Ethical approval for the study was obtained from the Cambridge Local Research Ethics Committee (REC number: 06/Q0108/161). Control participants were recruited under ethical approvals from the West London and Gene Therapy Advisory Committee National Research Ethics Service committee (11/H0707/9), and the Cambridge Local Research Ethics Committee (06/Q0108/303).

### 2.2 Assessment protocol

Following informed consent, assessments of boxers were performed on three occasions. The first, baseline assessment was completed prior to commencing sparring or training. The second, post-bout assessment was within 48 h after the first competitive bout. The final, one-year follow-up assessment was at approximately one year after the post-bout assessment.

### 2.3 Imaging parameters

Imaging was performed a Siemens Tim Trio operating at 3T at the Wolfson Brain Imaging Centre, University of Cambridge, UK.

A T1-weighted image was acquired with the MPRAGE sequence, with parameters: acquisition matrix size 256 × 256; field of view 256 mm × 256 mm; 1 mm slice thickness; repetition time, TR = 2300 ms and; echo time, TE = 2.98 ms.

Images sensitive to water diffusion were acquired in 63 non-collinear directions with b = 1000s/mm^2^, and two images without diffusion weighting, b = 0 s/mm^2^. Each image was acquired parallel to anterior-posterior (AC-PC) commissural line using an echo-planar imaging (EPI) sequence, with parameters: matrix size 96 × 96; field of view 192 mm × 192 mm; 63 axial slices; slice thickness = 2 mm; TR = 7800 ms, TE = 90 ms.

Prior to data analysis, MRI scans were viewed independently by two consultant neuroradiologists, one blinded to the timing of the scan (i.e. baseline, post-bout or one year follow-up), and the other aware of the scan timing. Additional sequences available for clinical assessment included gradient echo, FLAIR, and T2.

### 2.4 Neuroimaging analyses

Methods are described briefly below: full details are presented in the Supplemental information. Cortical thickness was estimated in two ways, with each method undertaking dissimilar approaches to image processing of T1-weighted MRI. Automated volume based cortical thickness estimation was performed with Advanced Normalisation Tools (ANTS, http://www.stnava.github.io) (Avants et al., 2009, Tustison et al., 2013, Das et al., 2009) while surface based estimation of cortical thickness estimation was performed with the FreeSurfer image analysis suite (http://surfer.nmr.mgh.harvard.edu/) (Reuter et al., 2010, Ségonne et al., 2004, Fischl et al., 2002, Fischl et al., 2004, Sled et al., 1998, Fischl et al., 2001, Ségonne et al., 2007, Dale et al., 1999, Fischl and Dale, 2000, Dale and Sereno, 1993).

Estimates of grey and white matter volumes at each intracerebral location were from T1-weighted MRI with FSL-VBM (http://fsl.fmrib.ox.ac.uk/fsl/fslwiki/FSLVBM (Douaud et al., 2007)), an optimized VBM protocol (Good et al., 2001) carried out with FSL tools (Smith et al., 2004, Andersson et al., 2008a).

Voxelwise statistical analysis of white matter DTI metrics, specifically FA and MD data, was carried out using Tract Based Spatial Statistics (TBSS) (Smith et al., 2006a), part of FSL (Smith et al., 2004, Smith et al., 2006b, Andersson et al., 2008b, Rueckert et al., 1999)

### 2.5 Neuropsychological assessment

Participants were asked to perform a series of neuropsychological tests from the Cambridge Neuropsychological Test Automated Battery (CANTAB, Cambridge Cognition, Cambridge, United Kingdom; http://www.cambridgecognition.com). The tests were computerized and run on a Paceblade touch-screen computer with responses registered via touch-screen or a button box. Participants were given the tests outlined in Table 1, in the order in which they are listed.

**Table 1:** Neuropsychological assessments.

Test	Measures	Outcome measures	Population norm (mean, SD)^a^	Sample (mean, SD)
Rapid visual information processing (RVP) (Park et al., 1994)	Sustained attention	• RVP A′	0.94, 0.03	0.92, 0.04
Paired associates learning (PAL) (Blackwell et al., 2004)	Learning and memory	• Total errors	0.92, 1.19	1.92, 3.05
Stop signal task (SST) (Aron et al., 2003)	Processing speed Response inhibition	• Go reaction time • Stop signal reaction time	329, 61 ms 211, 49 ms	426, 55 ms 161, 33 ms
One touch stockings of Cambridge (OTS) (Owen et al., 1995)	Planning Latency to problem solve	• Mean attempts to correct • Latency to correct response	1.24, 0.01 241.20, 77.39 ms	7.70, 1.79 179.31, 60.70 ms
Digit span (Lezak et al., 1995, Wechsler, 1981)	Verbal working memory capacity Verbal working memory and reordering	• Forward score • Backward score	9.75, 1.83 8.67, 2.01	9.60, 2.62 8.65, 2.41

a Population norms are not matched for NART.

### 2.6 Statistical testing

Case-control differences at baseline in grey and white matter volume were initially tested across the entire cerebral cortex and cerebellum (i.e. whole-brain). Then, within the group of boxers, acute changes in grey and white matter volumes, FA and MD, and the two estimates of cortical thickness were tested by paired comparison of data acquired at baseline and post-bout assessments. Long-term changes were similarly made by comparison of data acquired at baseline and one-year (post-bout) follow-up.

All imaging data was analyzed using a voxelwise general linear model (GLM), unpaired for baseline case-control comparisons, and paired for within-group longitudinal comparisons. Statistical inference was with the Randomize software using permutation-based (10,000 permutations), non-parametric testing and threshold free cluster enhancement (TFCE), correcting for the multiple comparisons associated with this mass-univariate approach by the family wise error rate (FWER). The corrected threshold for significance was p < 0.05. To undertake testing on cortical thickness estimated by FreeSurfer, surface ribbons were converted to volumes.

Baseline psychological data from the neuropsychological assessments were compared to the internal normative database of CANTAB (involving 3000 healthy volunteers matched for gender and age) or from other publications (Turner et al., 2003, Müller et al., 2013). Longitudinal testing within the group of boxers for acute and long-term changes were analyzed with SPSS version 10.0 for Windows (SPSS Inc., Chicago IL) with the same approach as for the imaging data; namely, baseline compared to post-bout, and baseline compared to one-year follow-up. The threshold for significance was p < 0.05, two-tailed.

## 3 Results

### 3.1 Participants

**Fig. 1:**
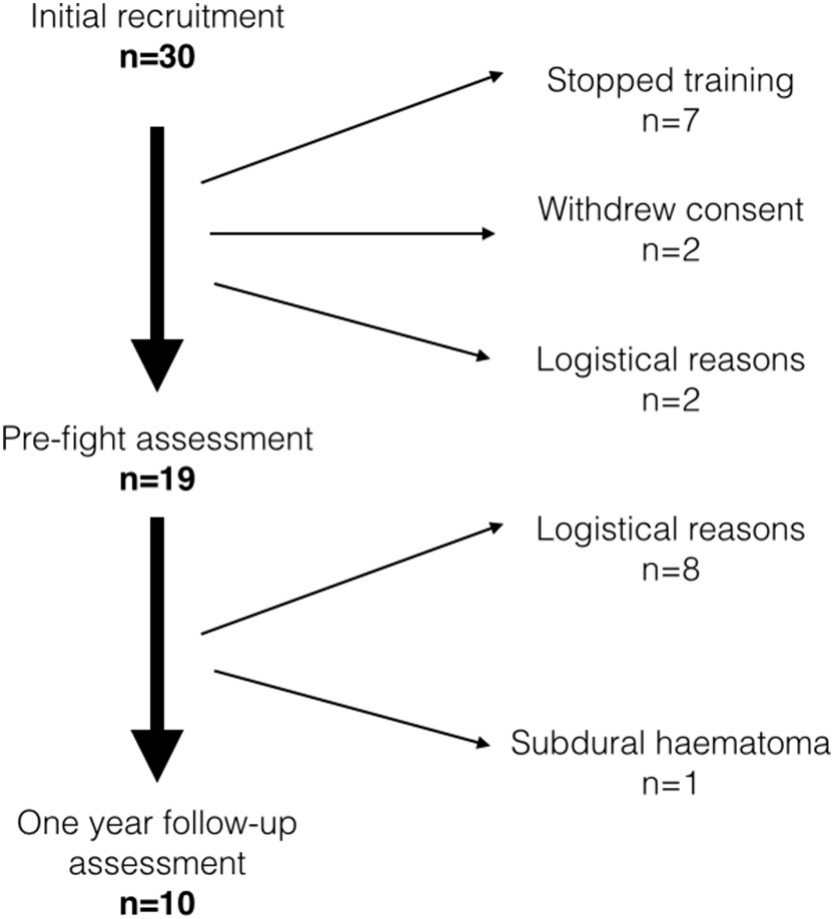
Recruitment flow chart. Reasons and numbers for the withdrawal of study participants prior to each assessment time point. Logistical reasons includes participants that finished university and moved away, therefore they were unable to return for assessment prior to the study closing.

A total of 30 boxers were recruited of which 10 (1 female, Mean age was 22.0 ± 2.3 years) completed the final assessments, and were included in the subsequent analyses. Reasons for withdrawal are illustrated in the flow chart in Fig. 1. Logistical reasons included participants that were lost to follow-up as well as others that had completed their studies in Cambridge and had moved away, and thus were unavailable for follow-up. Baseline demographic data is displayed in Table 2. The mean time between baseline and post-bout assessments was 37.8 ± 35.8 days (range: 5–95 days), and between the post-bout and one-year follow-up assessments was 460.6 ± 102.9 days (range: 362–628 days).

**Table 2:** Participant demographics. Values given are means (standard deviation).

	Recruited (n = 30)	Included in analysis (n = 10)
Age	22.2 (2.2) years	22.0 (2.3) years
Gender	28 male: 2 female	9 male: 1 female
Intelligence quotient (IQ), NART	117.4 (5.9)	119.0 (3.8)

Structural T1-weighted MRI were contemporaneously acquired from 10 control participants (1 female) with similar mean age of 22.0 ± 2.3 years.

### 3.2 Clinical events

Two clinical events occurred in the study period. One boxer developed seizures that were successfully controlled with anti-convulsants, while another boxer developed a chronic subdural haematoma requiring drainage. Both events occurred in the interval between the first fight and one-year follow-up, during a period when neither of them was actively engaged in sparring, nor had they recently participated in a bout. Although both participants stopped boxing, they both completed their one-year follow-up scans. Subsequently their management was overseen locally and no further long-term follow-up data is available. In both participants, all neuroimaging and neurocognitive data was normal, both prior and subsequent to the clinical events, apart from extra-cerebral post-surgical artefacts observed at time point 3 in the participant who had undergone drainage of the subdural haematoma.

### 3.3 Clinical MRI reporting

No structural brain abnormalities were detected on MRI from the boxers over the study period.

### 3.4 Neuroimaging analyses

No significant whole-brain, case-control differences changes were observed at baseline for grey or white matter, or estimates of cortical thickness from FreeSurfer or ANTS. Similarly, no significant whole-brain, acute or longer-term longitudinal differences in the boxers were observed with the neuroimaging estimates of grey and white matter, FA or MD, or estimates of cortical thickness.

### 3.5 Neuropsychological assessments

Participants performed, on average, equal to, or better than equivalent population norms (Table 1), which is consistent with a sample of university undergraduates compared to the general population.

**Fig. 2:**
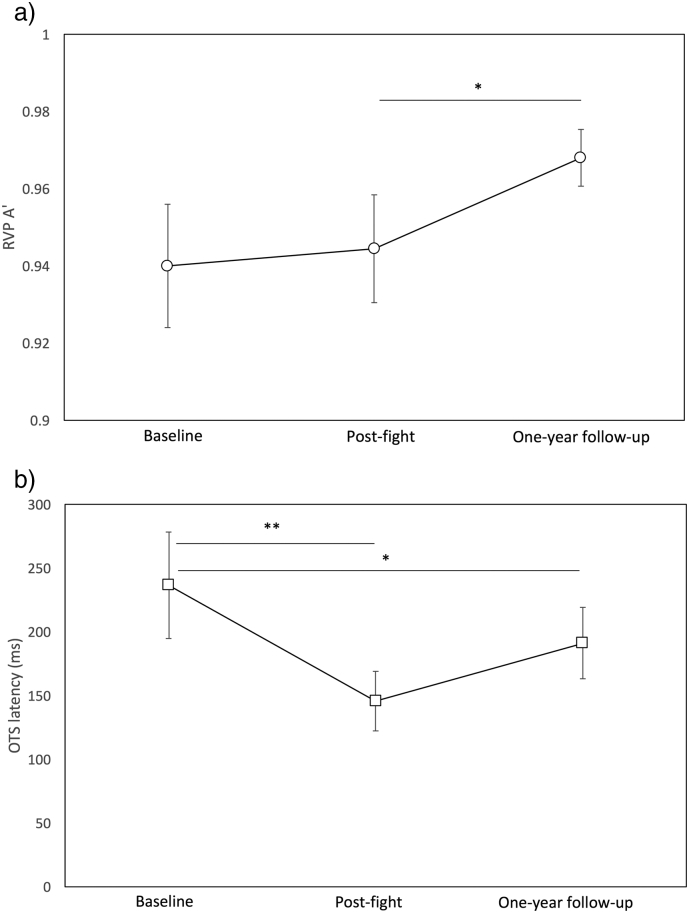
Neuropsychology findings: Mean and standard deviations at each assessment for (top) rapid visual information processing (RVP) A′ and (bottom) One Touch Stockings of Cambridge (OTS) latency to correct response. *p < 0.05 and **p < 0.01.

Paired *t*-tests between baseline and post-bout assessments were significant for the Rapid Visual Information Processing (RVP) A′ (*t*(9) = 2.3, p = 0.050) and One Touch Stockings of Cambridge (OTS) latency to correct response (*t*(9) = 4.44, p = 0.002), and between baseline and one year follow-up assessments for OTS latency to correct response (*t*(9) = 2.59, p = 0.016); Fig. 2a and b. All other tests were non-significant. A Bonferroni correction of the 16 tests undertaken renders only the OTS latency to correct response at baseline and post-bout assessments as significant.

## 4 Discussion

Out of 10 boxers followed up for over 1 year, one boxer developed seizures and another developed a chronic subdural haematoma requiring neurosurgical drainage. However, there was no evidence of acute or long-term brain injury using measures of grey and white matter volume, cortical thickness, or white matter microstructure. Neuropsychological assessment indicated that the sample followed were within (or indeed exceeded) normal ranges at baseline, compared to population norms (Table 1). Subsequently, attention tended to increase over all time points while planning and problem solving latency decreased after a bout, but showed some recovery at one-year follow-up.

The neuropsychological findings could be interpreted as successful boxing improving concentration in order to avoid being hit by the opponent or as potential beneficial effects of exercise (Sahakian, 2014). Alternatively, they may reflect a practice effect of repeating the tasks. Unfortunately our study design, which did not include longitudinal control group data, is unable to resolve this issue. Overall these findings should be taken in the context of a lack of changes in the neuroimaging data and the majority of the neuropsychological tasks.

### 4.1 Neuroimaging in sport

Combining advanced neuroimaging and neuropsychological testing has played a significant role in improving our understanding of repetitive brain injury during sport. The majority of research has involved participants in American football and ice hockey, while neuroimaging methods have included diffusion tensor and kurtosis imaging, magnetic resonance spectroscopy, task based and resting state functional MRI, transcranial magnetic stimulation, and electroencephalography. Such studies complement post-mortem reports (the traditional workhorse for understanding cumulative effects of brain injury) by allowing non-invasive, affordable longitudinal follow-up and correspondence with neuropsychological function in vivo.

### 4.2 Neuroimaging in boxing

Research into boxing related brain injury has spanned over 30 years and included a variety of neuroimaging techniques. Findings have generally been mixed with many studies failing to detect significant signs of brain injury or only finding non-specific abnormalities such as cerebral atrophy or a cavum septum pellucidum (Levin et al., 1987, Jordan and Zimmerman, 1988, Haglund and Bergstrand, 1990, Holzgraefe et al., 1992, Hähnel et al., 2008, Hasiloglu et al., 2011, Jordan and Zimmerman, 1990b). However, the use of DTI allowed the first consistently demonstrable abnormalities in professional boxers. Initially findings highlighted deranged whole brain average diffusivity (Zhang et al., 2003), but subsequently localized changes were identified in the corpus callosum (Zhang et al., 2006), and ultimately multivariate methods demonstrated additional changes in the inferior temporal lobes and internal capsule too (Chappell et al., 2008). These studies have served as a proof of principle that modern advanced neuroimaging methods have sensitivity to detect hitherto unidentified pathological findings.

One of the challenges of detecting subtle neuroimaging abnormalities is to determine what neuropsychological inference can be made. Recently regional DTI metrics (specifically FA and MD) have been found to correlate with neuropsychological function (declarative memory and reaction times) and boxing exposure (years of boxing) in a small cohort study, but corrections for multiple comparisons were not applied (Wilde et al., 2015). The large Professional Fighters Brain Health Study from Las Vegas has generated numerous publications in their large cohort of boxers and mixed martial art fighters. This study also found DTI metrics to correlate with neuropsychological function (reaction times) (Bernick et al., 2015b) and boxing exposure (number of knockouts) (Shin et al., 2014). Additionally, volumetric analysis of subcortical structures (specifically the caudate and thalamus) was found to correlate with cognitive impairment, slower processing, and fight exposure (Bernick et al., 2015a). Together these findings suggest that the findings identified with advanced neuroimaging methods are biologically and clinically meaningful, as well as reproducible.

A significant methodological concern with all these studies is case selection bias, with studies typically focusing on retired professional boxers with gross neuropsychological deficits, rather than including a representative contemporary consecutive series of boxers. Other limitations of the existing literature include performing cross sectional rather than longitudinal analysis and a lack of objective definitions for reporting abnormalities.

### 4.3 Strengths of this study

This study used high-resolution MRI of brain structure, and multiple established-methods for detecting local changes in grey and white matter and cortical thickness, as well as DTI to depict measures of white matter microstructure. All these methods have been used extensively to characterise a wide variety of neurological and psychiatric disorders. For each of these methods the statistical methodology for inference was equivalent, and appropriate corrections were made to the threshold for significance to account for multiple comparisons. In short, a rigorous approach to image processing and statistical testing did not reveal any consistently located longitudinal changes in brain structure of a small group of amateur boxers who were not significantly different from non-boxing controls at baseline.

A major strength of our study is the prospective cohort design, which is unique within boxing studies, and provides a valuable opportunity to monitor for brain injury developing over time. This has the dual benefit of helping to establish a causal link between imaging changes and boxing exposure while additionally it can be used to improve the safety of the participants if it detects early signs of brain injury. Recruiting from university boxing club has numerous advantages including: enrolment of previously healthy participants that had not boxed prior to entering the study; aids in establishing uniformity baseline demographics; and minimises confounding factors such as training regimes and competition intensity.

### 4.4 Potential limitations of the study design

Difficulties were encountered in the recruitment of boxers from a university amateur boxing club and included: limited follow-up due to participants finishing university or stopping boxing due to exams; possibly a reduced intensity of fighting and training; concordant changes in brain development and structure during the typical age range. On average, we managed to recruit approximately 10 new boxers per year, with around half of these continuing the sport and training regularly. More detailed recording of fighting demographics – such as frequency of training, number of fights, number of punches taken and total knockouts – would have been useful to correlate imaging changes with exposure to injury. Additionally novel MRI sequences, such as susceptibility weighted imaging, may offer improved sensitivity to detecting micro-haemorrhages.

Absence of evidence is not, in this case, evidence of absence of abnormalities in our cohort. False-negative errors may have arisen due to the small sample, the young age and good health of our participants over a relatively short follow-up period, or that our cohort did not sustain many blows to the head during training or bouts, although this was not directly recorded. Furthermore, the imaging methods used for statistical comparison assume the co-location of any changes, whereas brain injury can vary depending on the type of impact received.

## 5 Conclusion

Our method of establishing an advanced MRI protocol together with automated neuropsychological testing provides a novel contribution to the debate on boxing safety. While we did not find neuroimaging or neurocognitive evidence of brain injury in amateur boxers with this protocol, the clinical findings raise concern with one participant developing a chronic subdural haematoma requiring drainage and another developing seizures. The safety of amateur boxing requires further investigation.

## Funding

This study was supported through the Cambridge National Institute for Health Research (NIHR) (RRZB/003) Biomedical Research Centre (BRC). Control data were acquired with the support of the Medical Research Council as part of their Addiction Initiative (grant number G1000018), and a Pathfinder award from Medical Research Council (G0401099).

## Competing interests

Nil to declare.

## Appendix A Supplementary data

Supplementary material Image 1
