# Supplementary material for: Advanced magnetic resonance imaging and neuropsychological assessment for detecting brain injury in a prospective cohort of university amateur boxers
Source: Neuroimage Clin. 2017 Apr 26;15:194–9. doi: 10.1016/j.nicl.2017.04.026 (PMC5429235; doi:10.1016/j.nicl.2017.04.026)
Supplement: Supplementary material [file mmc1.docx]

**Estimation of Cortical Thickness**

Cortical thickness was estimated in two ways, with each method undertaking dissimilar approaches to image processing of T1-weighted MRI.

Advanced Normalisation Tools (ANTS) used an automated volume based cortical thickness estimation workflow according to the following steps: 1) initial N4 bias correction on input anatomical MRI; 2) brain extraction using a hybrid segmentation/template-based strategy; 3) alternating between prior-based segmentation and white matter posterior probability weighted bias correction; 4) DiReCT-based cortical thickness estimation; and 5) normalization to a group specific template

(Avants, Tustison and Johnson, n.d.; Tustison, Avants and Cook, 2013; Das, Avants, Grossman and Gee, 2009).

Cortical reconstruction and thickness estimation was performed with the FreeSurfer image analysis suite (http://surfer.nmr.mgh.harvard.edu/). Briefly, processing included: 1) motion correction and averaging of multiple volumetric T1 weighted images (when more than one was available) (Reuter, Rosas and Fischl, 2010); 2) removal of non-brain tissue using a hybrid watershed/surface deformation procedure (Ségonne et al., 2004); 3) automated Talairach transformation, segmentation of the subcortical white matter and deep gray matter volumetric structures (including hippocampus, amygdala, caudate, putamen, ventricles) (Fischl et al., 2002; 2004); 4) intensity normalization (Sled, Zijdenbos and Evans, 1998); 5) tessellation of the gray matter white matter boundary, automated topology correction (Fischl, Liu and Dale, 2001; Ségonne, Pacheco and Fischl, 2007); and 5) surface deformation following intensity gradients to optimally place the gray/white and gray/cerebrospinal fluid borders at the location where the greatest shift in intensity defines the transition to the other tissue class (Dale, Fischl and Sereno, 1999; Dale and Sereno, 1993; Fischl and Dale, 2000).

**Voxel Based Morphometry**

Estimates of grey and white matter volumes at each intracerebral location were from T1-weighted MRI with FSL-VBM (<http://fsl.fmrib.ox.ac.uk/fsl/fslwiki/FSLVBM> (Douaud et al., 2007) an optimized VBM protocol (Good et al., 2001) carried out with FSL tools (Smith et al., 2004). First, structural images were brain-extracted and grey matter-segmented before being registered to the MNI 152 standard space using non-linear registration

(Andersson, Jenkinson and Smith, n.d.). The resulting images were averaged and flipped along the x-axis to create a left-right symmetric, study-specific grey matter template. Second, all native grey matter images were non-linearly registered to this study-specific template and "modulated" to correct for local expansion (or contraction) due to the non-linear component of the spatial transformation. The modulated grey matter images were then smoothed with an isotropic Gaussian kernel with a sigma of 2 mm.

**Tract Based Spatial Statistics (TBSS)**

Pre-statistical processing of diffusion images included eddy current correction, motion correction, and averaging of the three sets of 63 diffusion directions using software from FMRIB’s Diffusion Toolbox, FDT (Smith et al., 2004). A brain mask produced using the Brain Extraction Tool (Smith, 2002) (BET) along with the diffusion data, was used by DTIFit(Smith et al., 2004) to calculate the 3x3 diffusion tensor for each brain voxel, and subsequently compute fractional anisotropy (FA) and mean diffusivity (MD) from the tensor’s three eigenvalues.

Voxelwise statistical analysis of the FA and MD data was carried out using TBSS (Smith et al., 2006), part of FSL (Smith et al., 2004). All participant’s FA images were aligned into the standard anatomical space of the Montreal Neurological Institute (MNI) using the non-linear registration tool FNIRT

(Andersson, Jenkinson and Smith, n.d.; n.d.), which used a b-spline representation of the registration warp field (Rueckert et al., 1999). This same transform was subsequently applied to the MD images. Next, the mean FA image was created and thinned to create a mean FA skeleton that represented the centres of all tracts common to the group. The mean WM skeleton was then thresholded to include only those voxels with FA > 0.3, which excludes regions of high between-subject variability. Each participant’s aligned FA image was then projected onto this skeleton, with an identical procedure for the MD images.

**REFERENCES**

Andersson, J.L., Jenkinson, M. and Smith, S., n.d. Non-linear optimisation. *FMRIB technical report TRJA*. [online] Available at: <www.fmrib.ox.ac.uk/analysis/techrep>.

Andersson, J.L., Jenkinson, M. and Smith, S., n.d. Non-linear registration, aka Spatial normalisation. *FMRIB technical report TRJA*. [online] Available at: <www.fmrib.ox.ac.uk/analysis/techrep

Avants, B.B., Tustison, N. and Johnson, H., n.d. *Advanced normalization tools (ANTS) 2009*. *Insight J*

Dale, A.M. and Sereno, M.I., 1993. Improved Localizadon of Cortical Activity by Combining EEG and MEG with MRI Cortical Surface Reconstruction: A Linear Approach. *Journal of Cognitive Neuroscience*, 5(2), pp.162–176.

Dale, A.M., Fischl, B. and Sereno, M.I., 1999. Cortical surface-based analysis. I. Segmentation and surface reconstruction. *NeuroImage*, 9(2), pp.179–194.

Das, S.R., Avants, B.B., Grossman, M. and Gee, J.C., 2009. Registration based cortical thickness measurement. *NeuroImage*, 45(3), pp.867–879.

Douaud, G., Smith, S., Jenkinson, M., Behrens, T., Johansen-Berg, H., Vickers, J., James, S., Voets, N., Watkins, K., Matthews, P.M. and James, A., 2007. Anatomically related grey and white matter abnormalities in adolescent-onset schizophrenia. *Brain: a Journal of Neurology*, 130(Pt 9), pp.2375–2386.

Fischl, B. and Dale, A.M., 2000. Measuring the thickness of the human cerebral cortex from magnetic resonance images. *Proceedings of the National Academy of Sciences*, 97(20), pp.11050–11055.

Fischl, B., Liu, A. and Dale, A.M., 2001. Automated manifold surgery: constructing geometrically accurate and topologically correct models of the human cerebral cortex. *IEEE transactions on medical imaging*, 20(1), pp.70–80.

Fischl, B., Salat, D.H., Busa, E., Albert, M., Dieterich, M., Haselgrove, C., van der Kouwe, A., Killiany, R., Kennedy, D., Klaveness, S., Montillo, A., Makris, N., Rosen, B. and Dale, A.M., 2002. Whole brain segmentation: automated labeling of neuroanatomical structures in the human brain. *Neuron*, 33(3), pp.341–355.

Fischl, B., Salat, D.H., van der Kouwe, A.J.W., Makris, N., Ségonne, F., Quinn, B.T. and Dale, A.M., 2004. Sequence-independent segmentation of magnetic resonance images. *NeuroImage*, 23 Suppl 1, pp.S69–84.

Good, C.D., Johnsrude, I.S., Ashburner, J., Henson, R.N.A., Friston, K.J. and Frackowiak, R.S.J., 2001. A Voxel-Based Morphometric Study of Ageing in 465 Normal Adult Human Brains. *NeuroImage*, 14(1), pp.21–36.

Reuter, M., Rosas, H.D. and Fischl, B., 2010. Highly accurate inverse consistent registration: a robust approach. *NeuroImage*, 53(4), pp.1181–1196.

Rueckert, D., Sonoda, L.I., Hayes, C., Hill, D.L., Leach, M.O. and Hawkes, D.J., 1999. Nonrigid registration using free-form deformations: application to breast MR images. *IEEE transactions on medical imaging*, 18(8), pp.712–721.

Ségonne, F., Dale, A.M., Busa, E., Glessner, M., Salat, D., Hahn, H.K. and Fischl, B., 2004. A hybrid approach to the skull stripping problem in MRI. *NeuroImage*, 22(3), pp.1060–1075.

Ségonne, F., Pacheco, J. and Fischl, B., 2007. Geometrically accurate topology-correction of cortical surfaces using nonseparating loops. *IEEE transactions on medical imaging*, 26(4), pp.518–529.

Sled, J.G., Zijdenbos, A.P. and Evans, A.C., 1998. A nonparametric method for automatic correction of intensity nonuniformity in MRI data. *IEEE transactions on medical imaging*, 17(1), pp.87–97.

Smith, S.M., 2002. Fast robust automated brain extraction. *Human Brain Mapping*, 17(3), pp.143–155.

Smith, S.M., Jenkinson, M., Johansen-Berg, H., Rueckert, D., Nichols, T.E., Mackay, C.E., Watkins, K.E., Ciccarelli, O., Cader, M.Z., Matthews, P.M. and Behrens, T.E.J., 2006. Tract-based spatial statistics: voxelwise analysis of multi-subject diffusion data. *NeuroImage*, 31(4), pp.1487–1505.

Smith, S.M., Jenkinson, M., Woolrich, M.W., Beckmann, C.F., Behrens, T.E.J., Johansen-Berg, H., Bannister, P.R., De Luca, M., Drobnjak, I., Flitney, D.E., Niazy, R.K., Saunders, J., Vickers, J., Zhang, Y., De Stefano, N., Brady, J.M. and Matthews, P.M., 2004. Advances in functional and structural MR image analysis and implementation as FSL. *NeuroImage*, 23 Suppl 1, pp.S208–19.

Tustison, N.J., Avants, B.B. and Cook, P.A., 2013. The ANTs cortical thickness processing pipeline. *Proceeds of SPIE*, 8672, pp.86720K–2.
